# Supplementary material for: Immunophenotypic Alterations in Adult Patients with Steroid-Dependent and Frequently Relapsing Nephrotic Syndrome
Source: Int J Mol Sci. 2023 Apr 22;24(9):7687. doi: 10.3390/ijms24097687 (PMC10178237; doi:10.3390/ijms24097687)
Supplement: Supplementary file 1 [file ijms-24-07687-s001.zip › ijms-2290796-supplementary.pdf]

Supplementary Table S1. Percentages of $CD8^+$ and $CD4^+$ T cell subsets in SDNS/FRNS and SRNS patients and in healthy controls

	**SDNS/FRNS**	**SRNS**	**HV**	**P value**
	(n=18)	(n=7)	(n=15)	
**$CD8^+$ T cells**				
Naïve	6.061 [1.95-12.01]	8.350 [6.94-10.97]	4.147 [1.67-9.86]	NS
TCM	0.306 [0.07-0.48] *	0.080 [0.05-0.36] *	0.676 [0.36-1.04]	P<0.05 vs HV
$CD28^+$ TEM	7.254 [4.96-1082]	5.660 [4.53-10.12]	9.933 [6.91-14.17]	NS
$CD28^-$ TEM	2.286 [0.72-3.97]	1.370 [0.67-1.92]	2.910 [1.27-6.57]	NS
TEMRA	10.419 [5.38-17.21]	10.700 [2.09-17.51]	17.590 [6.27-19.50]	NS
**$CD4^+$ T cells**				
Naïve	20.420 [12.88-31.23]	11.787 [8.83-14.95]	18.509 [13.48-22.14]	NS
TCM	4.342 [2.01-5.90]	5.304 [3.08-9.34]	7.567 [4.56-11.04]	NS
$CD28^+$ TEM	10.612 [7.24-19.15]	8.515 [7.30-13.63]	11.745 [7.68-16.25]	NS
$CD28^-$ TEM	0.161 [0.07-0.61]	0.973 [0.29-1.38]	0.260 [0.14-0.95]	NS

TCM: T central memory, TEM: T effector memory; TEMRA: CD45RA+ T effector memory.
Data are expressed as median % on parent cells [interquartile range]
* *p*<0.05 versus HV; NS: Not significant

Supplementary Table S2. Percentages of B cell subsets in relapsers and non-relapsers SDNS/FRNS patients at baseline before rituximab.

	**RELAPSERS**	**NON-RELAPSERS**	**P value**
	(n=9)	(n=9)	
**B cells**			
memory $CD27^{+}$	50.40 [42.65-61.98]	58.90 [31.88-66.03]	NS
IgM memory	24.39 [21.86-28.21]	24.63 [20.38-31.37]	NS
switched memory	15.61 [13.65-18.83]	16.50 [11.27-28.59]	NS
switched	19.15 [1.53-25.39]	17.96 [11.85-27.68]	NS
unswitched	24.43 [22.19-35.15]	30.22 [22.31-43.42]	NS
double negative	7.99 [6.56-11.20]	5.67 [5.00-7.74]	NS
naive	36.66 [32.46-50.23]	34.12 [24.75-62.85]	NS

Data are expressed as median % on parent cells [interquartile range]
NS: not significant

Supplementary Table S3. Percentages and counts of $CD8^+$ and $CD4^+$ T cell subsets in relapsers and non-relapser SDNS/FRNS patients at baseline before rituximab.

		**RELAPSERS**	**NON-RELAPSERS**	**P value**
		(n=9)	(n=9)	
**$CD8^+$ T cells**				
Naïve	% on parent cells	6.359 [4.89-13.41]	2.50 [0.60-11.96]	NS
	cells/microL	57.93 [37.13-128.31]	26.91 [6.14-117.02]	NS
TCM	% on parent cells	0.30 [0.10-0.48]	0.31 [0.05-0.49]	NS
	cells/microL	2.56 [0.83-3.75]	2.90 [0.54-4.25]	NS
CD28+ TEM	% on parent cells	6.19 [2.32-9.56]	9.25 [5.25-13.17]	NS
	cells/microL	40.42 [21.13-89.44]	82.16 [56.73-126.99]	NS
CD28- TEM	% on parent cells	1.65 [0.71-3.88]	2.34 [0.73-5.44]	NS
	cells/microL	8.63 [6.35-39.78]	25.23 [9.43-49.91]	NS
TEMRA	% on parent cells	9.625 [4.53-19.10]	14.78 [5.55-18.85]	NS
	cells/microL	46.64 [22.86-183.91]	98.14 [68.48-195.25]	NS
**$CD4^+$ T cells**				
Naïve	% on parent cells	20.58 [17.40-40.17]	17.06 [7.57-22.40]	NS
	cells/microL	319.67 [174.8-486.6]	215.77 [113.3-385.8]	NS
TCM	% on parent cells	3.94 [2.80-5.31]	4.75 [1.61-9.62]	NS
	cells/microL	49.14 [19.77-64.86]	70.28 [28.21-103.21]	NS
$CD28^+$ TEM	% on parent cells	8.08 [5.55-14.21]	18.71 [8.94-23.76]	NS
	cells/microL	90.90 [60.52-199.83]	196.36 [155.42-432.43]	NS
$CD28^-$ TEM	% on parent cells	0.15 [0.05-0.86]	0.20 [0.08-0.59]	NS
	cells/microL	1.36 [0.66-7.97]	3.64 [1.42-8.48]	NS

Data are expressed as median % on parent cells [interquartile range].
NS: not significant

Supplementary Table S4. B and T cell subsets in MCD and FSGS patients of the SDNS/FRNS patient group.

		MCD	FSGS	P value
		(n=11)	(n=7)	
**B cells**				
$CD19^{+}CD20^{+}$ B cells	% on lymphocytes	17.62 [10.23-28.56]	18.83 [18.37-19.55]	NS
memory	% on lymphocytes	10.21 [5.39-13.42]	11.05 [10.47-12.04]	NS
	% on B cells	54.86 [35.03-73.38]	57.73 [55.77-68.58]	NS
transitional	% on lymphocytes	0.05 [0.03-0.08]	0.08 [0.04-0.12]	NS
	% on B cells	0.20 [0.11-1.32]	0.40 [0.26-0.65]	NS
mature	% on lymphocytes	3.91 [2.38-7.42]	4.82 [2.42-4.93]	NS
	% on B cells	27.09 [13.09-41.93]	25.16 [12.73-26.42]	NS
memory $CD27^{+}$	% on lymphocytes	9.60 [3.70-11.52]	9.60 [7.45-10.96]	NS
	% on B cells	50.40 [31.82-61.82]	61.80 [45.38-65.72]	NS
IgM memory	% on lymphocytes	3.60 [2.45-6.93]	4.44 [4.09-5.33]	NS
	% on B cells	24.63 [14.84-30.93]	24.39 [23.20-27.66]	NS
switched memory	% on lymphocytes	2.48 [1.20-4.99]	2.86 [2.44-5.09]	NS
	% on B cells	14.65 [9.76-19.28]	15.96 [13.88-26.47]	NS
switched	% on lymphocytes	2.87 [1.21-5.25]	4.11 [3.05-5.98]	NS
	% on B cells	17.62 [9.76-19.83]	20.89 [15.46-36.34]	NS
unswitched	% on lymphocytes	4.18 [2.54-7.87]	5.37 [4.32-7.27]	NS
	% on B cells	30.08 [16.88-35.24]	27.29 [22.85-39.58]	NS
double negative	% on lymphocytes	1.31 [0.52-1.89]	1.23 [1.04-2.21]	NS
	% on B cells	6.74 [5.30-8.64]	6.80 [6.16-11.27]	NS
naive	% on lymphocytes	6.59 [6.00-12.96]	6.22 [3.86-8.86]	NS
	% on B cells	40.72 [32.39-63.51]	33.90 [22.15-47.44]	NS
**$CD4^{+}$ T cells**				
Tregs	% on $CD4^{+}$ T cells	0.286 [0.090-0.383]	0.108 [0.046-0.131]	NS
$CD45RO^{+}$ Tregs	% on $CD4^{+}$ T cells	0.088 [0.078-0.317]	0.026 [0.000-0.101]	NS
$CD45RA^{+}$ Tregs	% on $CD4^{+}$ T cells	0.000 [0.000-0.009]	0.006 [0.001-0.011]	NS
**$CD8^{+}$ T cells**				
Naïve	% on $CD8^{+}$ T cells	6.39 [2.90-11.99]	4.58 [1.23-10.41]	NS
TCM	% on $CD8^{+}$ T cells	0.44 [0.02-0.48]	0.30 [0.17-0.37]	NS
$CD28^{+}$ TEM	% on $CD8^{+}$ T cells	6.19 [5.05-11.59]	9.13 [3.46-10.69]	NS
$CD28^{-}$ TEM	% on $CD8^{+}$ T cells	1.65 [0.77-4.23]	3.05 [1.09-3.94]	NS
TEMRA	% on $CD8^{+}$ T cells	7.72 [5.19-13.49]	23.77 [12.11-30.37]	NS

Data are expressed as median [interquartile range].
NS: not significant

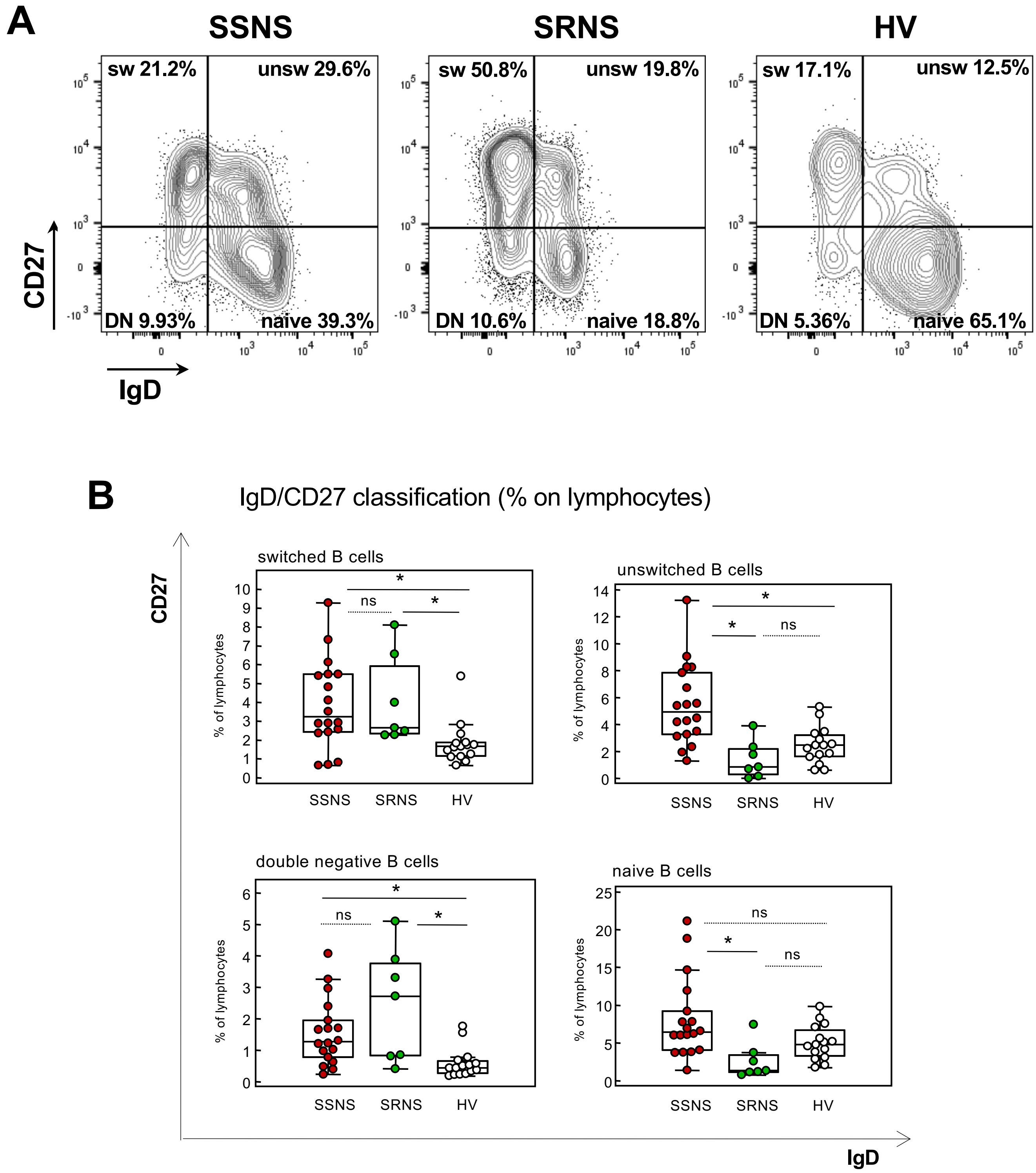

Figure S1. IgD/CD27 B cell subsets in SDNS/FRNS and SRNS patients and HV. (A) Representative contour plots of IgD/CD27 B cells in the three groups of subjects; **(B)** Percentages of switched, unswitched, double negative and naïve B cells among total lymphocytes in patients with SSNS or SRNS and in HV. Plots display the median, 25th and 75th percentiles of distribution (boxes) and whiskers extend to the minimum and maximum values of the series. *P<0.05 between the indicated groups. * p < 0.05 between the indicated groups, ns: not significant.

Figure S1

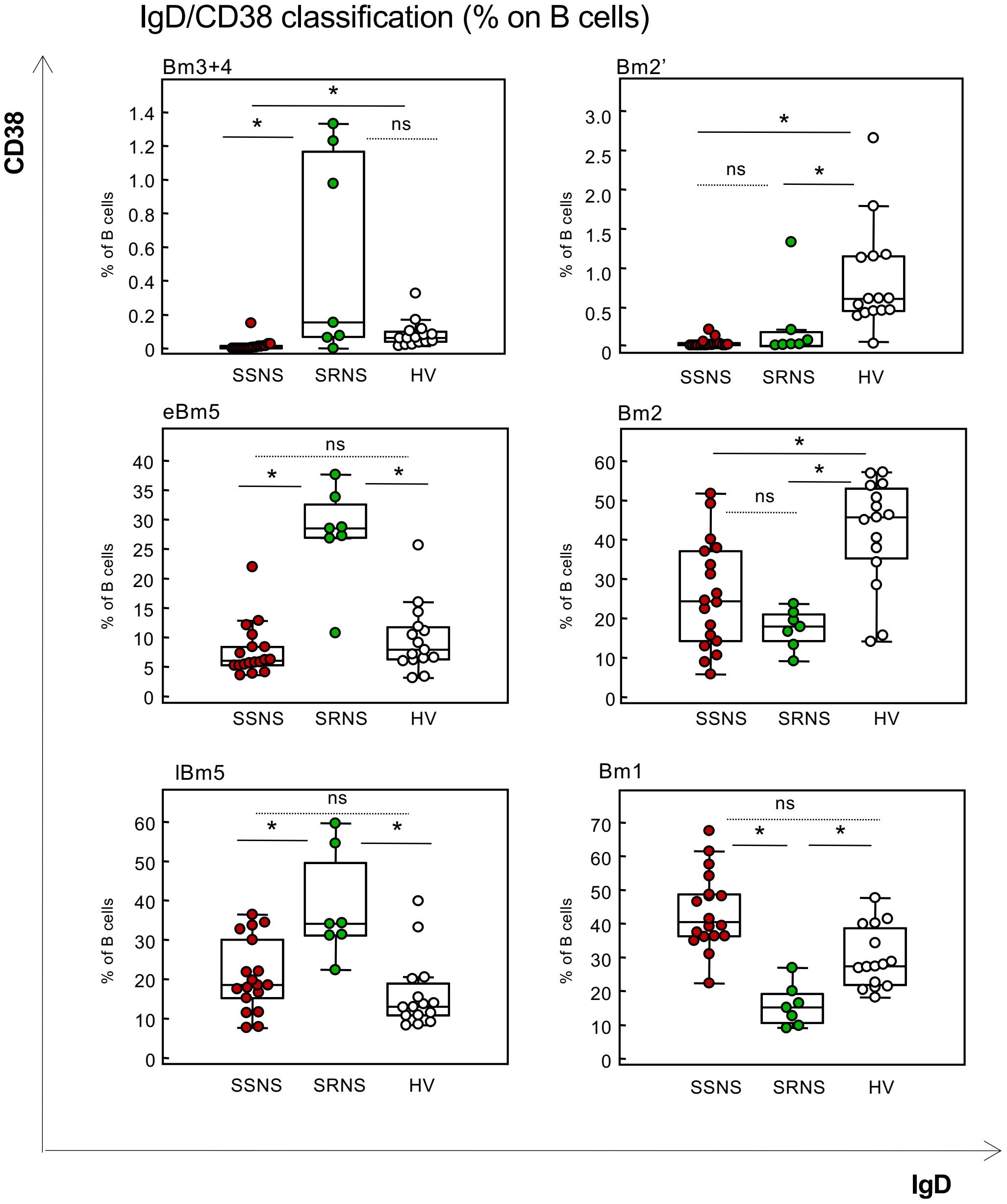

Figure S2. IgD/CD38 B cell subsets as percentages of B cells in SDNS/FRNS and SRNS patients and HV. Percentages of Bm1, Bm2, Bm2', Bm3+4, early and late Bm5 on $CD19^{+}CD20^{+}$ B cells in patients with SSNS or SRNS and in HV. Plots display the median, 25th and 75th percentiles of distribution (boxes) and whiskers extend to the minimum and maximum values of the series.
* *p* <0.05 between the indicated groups; ns: not significant.

Figure S2

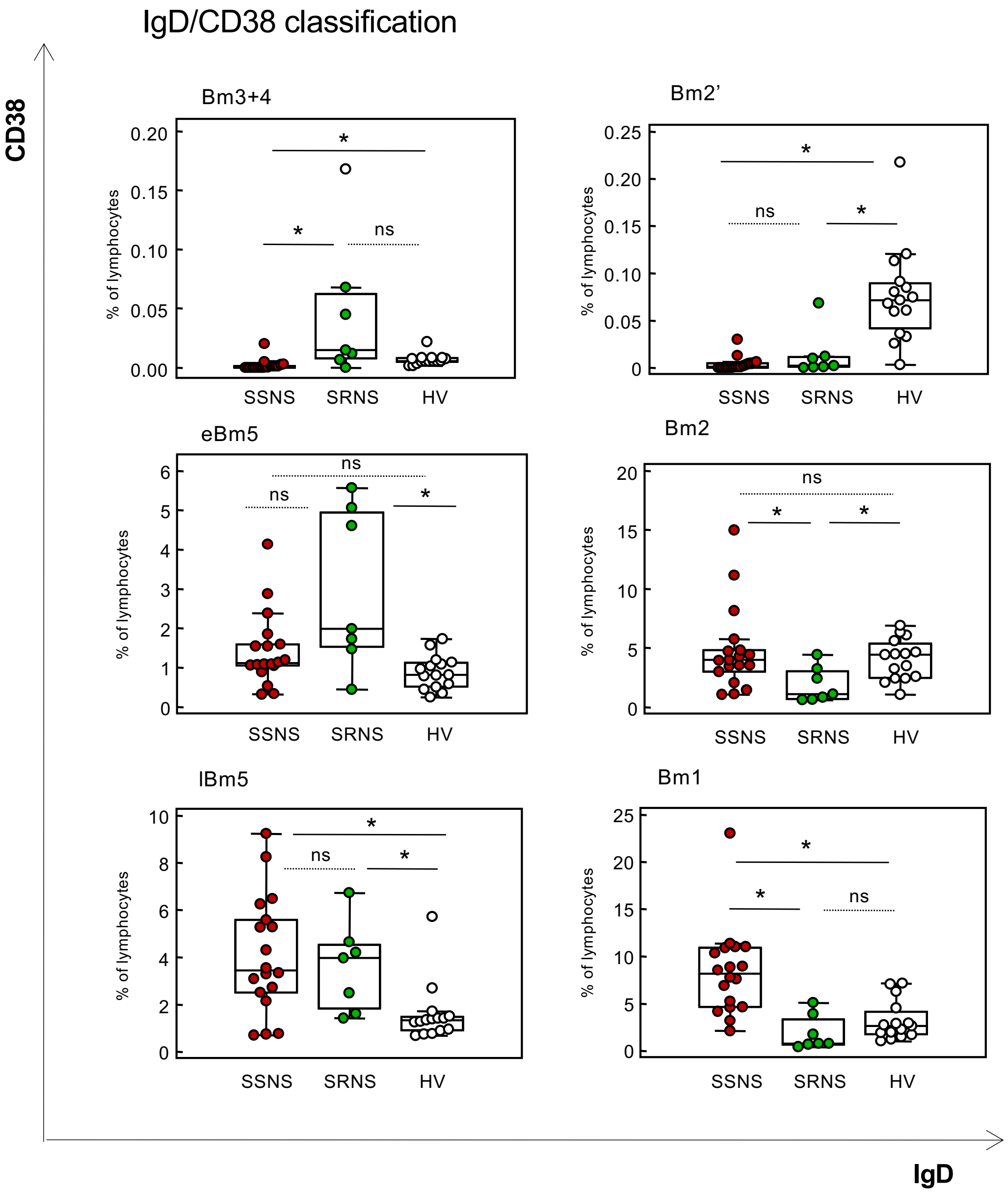

Figure S3. IgD/CD38 B cell subsets as percentages of total lymphocytes in SDNS/FRNS and SRNS patients and HV. Percentages of Bm1, Bm2, Bm2', Bm3+4, early and late Bm5 on total lymphocytes in patients with SSNS or SRNS and in HV. Plots display the median, 25th and 75th percentiles of distribution (boxes) and whiskers extend to the minimum and maximum values of the series. * $p < 0.05$ between the indicated groups, ns: not significant

Figure S3

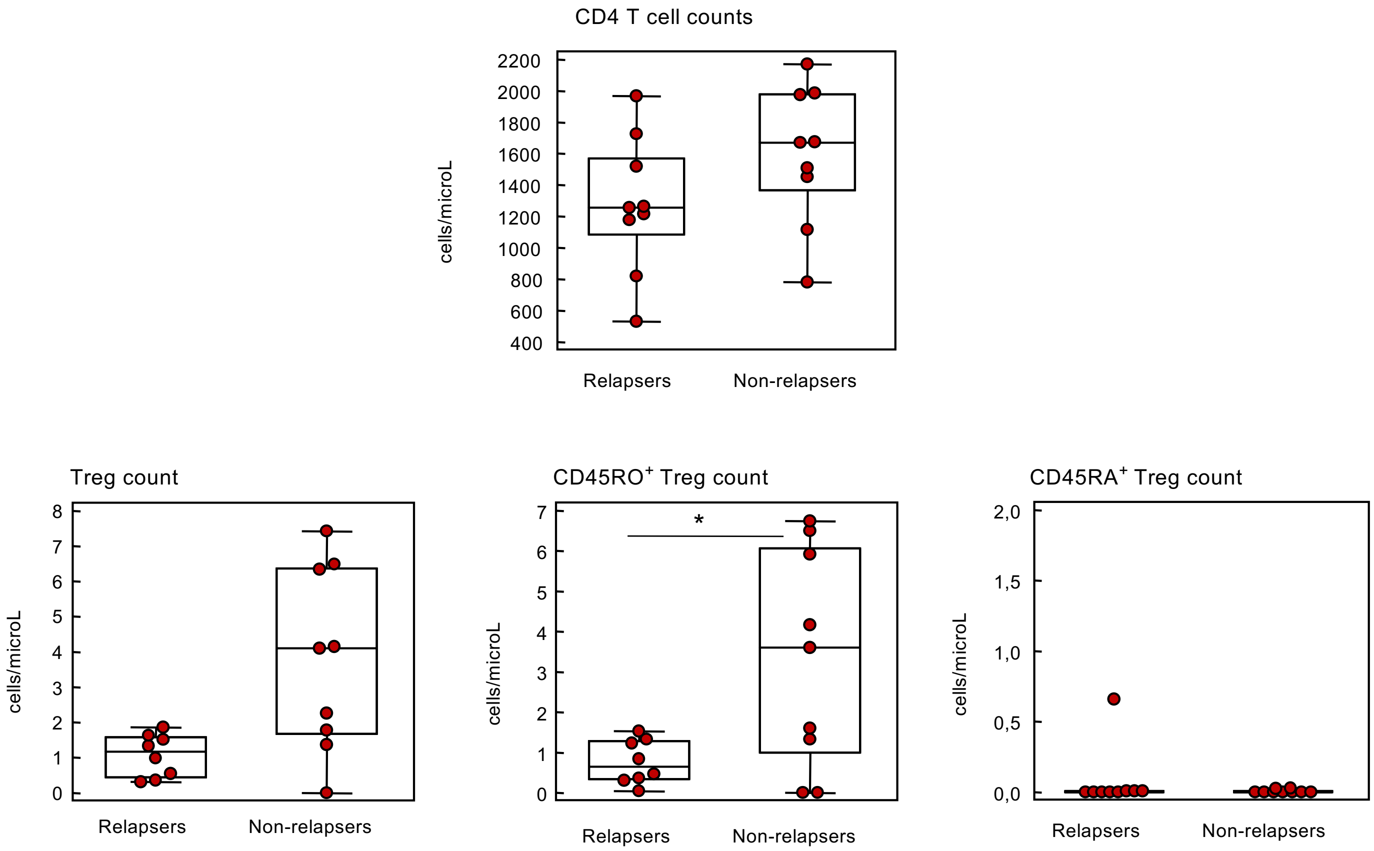

Figure S4. $CD4^{+}$ T cell and Treg counts in relapsers and non-relapsers SDNS/FRNS patients at baseline before rituximab. Counts of $CD4^{+}$ T cells and of total, memory and naive Tregs in SDNS/FRNS patients who relapsed or did not after rituximab therapy. Plots display the median, 25th and 75th percentiles of distribution (boxes) and whiskers extend to the minimum and maximum values of the series, * $p < 0.05$ between the indicated groups, ns: not significant.

Figure S4
